# A TCR mimic monoclonal antibody for the HPV-16 E7-epitope p11-19/HLA-A*02:01 complex

**DOI:** 10.1371/journal.pone.0265534

**Published:** 2022-03-17

**Authors:** Tao Dao, Sungsoo Mun, Tatyana Korontsvit, Abdul G. Khan, Mary Ann Pohl, Thomas White, Martin G. Klatt, David Andrew, Ivo C. Lorenz, David A. Scheinberg

**Affiliations:** 1 Molecular Pharmacology Program, Memorial Sloan Kettering Cancer Center, New York, New York, United States of America; 2 Tri-Institutional Therapeutics Discovery Institute, New York, New York, United States of America; 3 Weill Cornell Medicine, New York, New York, United States of America; National Cancer Institute, UNITED STATES

## Abstract

More effective treatments are needed for human papilloma virus (HPV)-induced cancers despite HPV virus vaccination. The oncogenic HPV protein targets are currently undruggable and intracellular and therefore there are no antibodies to these targets. Here we report the discovery of TCR mimic monoclonal antibodies (TCRm mAb) specific for the HPV E7 protein p11-19, YMLDLQPET, when presented on the cell surface in the context of HLA-A*02:01 by use of human phage display libraries. One of the mAbs, 3F8, was able to specifically mediate T cell- redirected cytotoxicity, in a bispecific T cell engager (BiTE) form. While further studies are required to assess the therapeutic potential of this approach, the study provided the proof of concept that TCRm mAb could be a therapeutic strategy for HPV-induced human cancers.

## Introduction

High risk HPV causes malignant transformation following persistent infection and is the cause of virtually all cases of cervical cancer and represents nearly 30% of all infection-related cancers [1]. The viral E6 and E7 proteins have transforming activities through functional inactivation of the p53 and retinoblastoma (Rb) tumor suppressor proteins, respectively [2,3]. Prophylactic vaccination-induced antibodies only neutralize virus particles before infections, but have no therapeutic efficacy for the cryptic oncogenic proteins remaining or the later induced cancers. HPV E6 and E7 viral oncogenic proteins are not currently druggable by small molecules and are intracellular and hence, inaccessible to therapeutic antibodies. T cells have been attributed to the natural clearance of most infected cells because T cells recognize and destroy the infected cells that present viral protein-derived peptide fragments complexed with HLA class I and class II molecules on the cancer cell surface. Therefore, T-cell based immunotherapies, such as vaccination or adoptive T cell transfer targeting viral-derived epitopes have been intensively explored for the treatment of HPV induced cancers recently [4–6].

HPV type 16 E6 and E7 proteins are consistently expressed in HPV-associated cancers and are thus ideal targets for vaccine design. Therefore, therapeutic vaccines have mostly focused on E6 and E7 as target antigens to induce HPV-specific T cell responses. Various formats of vaccines including naked DNA, short and overlapping long peptides, fusion constructs with Toll-like receptor agonists, have been studied. However, significant clinical benefit over historical controls remains to be observed [7–9]. Priming a broad T cell response by vaccination may only generate a small fraction of tumor lytic CD8+ T cells. Recently, adoptive transfer of TCR gene engineered T cells targeting HPV-16 epithelial cancer has also been explored [10,11], but this approach is patient-specific, which limits wide, cost effective application for most patients, especially outside of major cancer centers in developed countries.

We hypothesize that a mAb that mimics TCR recognition of a HPV-derived epitopes presented by HLA class I molecules could be an effective immunotherapeutic approach targeting HPV-induced malignancies. The advantages of mAb therapy are well known and include their high target specificity, high efficacy, limited side effects, prolonged half-life, availability, and infrequent dosing. These features make a mAb therapy particularly useful and practical in undeveloped countries. In addition to its inherent properties, mAb can be engineered into many formats to enhance its potency and not patient-specific as adoptive T cell therapy.

We selected a well-defined HPV-E7-derived CD8 T cell epitope [E7 p11-19, YMLDLQPET], as the target for the discovery of a TCRm mAb. This epitope has been reproducibly detected in most cervical cancer biopsies and HPV positive cancer cell lines in the context of HLA-A*02:01 molecule by mass spectrometry [12–14]. The epitope also has been shown to induces a CD8 T cell response in the context of HLA-A*02:01 molecule.

## Materials and methods

### Cell samples, cell lines, and antibodies

After written and witnessed informed consent on Memorial Sloan-Kettering Cancer Center [MSKCC] Institutional Review Board–approved protocols, peripheral blood mononuclear cells [PBMCs] from HLA-typed healthy donors were obtained by Ficoll density centrifugation. Tumor cell lines used in this study were obtained from American Tissue Culture Collection [ATCC]. mAbs against human HLA-A2 (clone BB7.2) conjugated to fluorescein isothiocyanate [FITC] or allophycocyanin (APC), and its isotype control mouse IgG2b/FITC or APC were purchased from Biolegend. Goat F(ab′)2 anti-hIgG conjugated with phycoerythrin [PE] or FITC, mouse anti-human CD3 mAb, and 6x-His Tag mAb/FITC were purchased from Invitrogen. APC conjugation kit-lighting link (ab201817) was purchased from Abcam and was used to label 3F8, 2A5 and 1B1 mAbs according to manufacturer’s instruction. Human isotype control hIgG1 antibody was purchased from Bingo Biotech [catalog number ET901].

### Peptides

All peptides were purchased and synthesized by Genemed Synthesis Inc (San Antonio, TX). Peptides were >95% pure (Table 1). The peptides were dissolved in dimethyl sulfoxide and diluted in saline at 5 mg/ml and frozen at −80°C.

**Table 1:** Peptides used for the selection and characterization of mAbs specific for HPV-E7p11/HLA-A2 complex.

**Native peptides**	**Sequences**
HPV-E7p11-19	YMLDLQPET
WT1-RMF	RMFPNAPYL
**Alanine substituted peptide names**	**Sequences**
A-1	***A***MLDLQPET
A-2	Y***A***LDLQPET
A-3	YM***A***DLQPET
A-4	YML***A***LQPET
A-5	YMLD***A***QPET
A-6	YMLDL***A***PET
A-7	YMLDLQ***A***ET
A-8	YMLDLQP***A***T
A-9	YMLDLQPE***A***
HPV-E7AAAA	YM***AAAA***PET

### Flow cytometry analysis

For cell surface staining, cells were incubated with appropriate mAbs for 30 min on ice, washed, and incubated with secondary antibody reagents when necessary. Flow cytometry data were collected on a LSR Fortessa (BD Biosciences) and analyzed with FlowJoV10.6.1 software.

### Production of HPV-E7p11/HLA-A2 peptide complexes

The method used follows directly the original established protocol [15–17]. Briefly, large amounts of soluble MHC class I/peptide complexes were generated by overexpression of HLA-A2 heavy chain (HC) and beta2 microglobulin (β2m) as recombinant proteins in *E*. *coli* and subsequent in vitro refolding and assembly in the presence of high concentrations of HPV-E7p11, WT1-RMF or HPV-AAAA peptide.

### Screening of phage library and engineering of full length human IgG1

A proprietary naïve, semi-synthetic human single chain fragment variable (scFv) phage display library [18] was used for the discovery of HLA/HPV-E7 p11-19 peptide complex specific clones. Two sub libraries HuScL-3 and HuScL-5 were enriched for phage that bound to the HLA-HPV-E7 peptide complex by three rounds of solution selection using standard phage display techniques. Briefly, prior to panning on HLA-E7 p11-19 peptide complex, the combined libraries were depleted with streptavidin beads followed by biotinylated HLA-irrelevant peptide complex at 10 ug/ml in blocking buffer for 30 minutes. The beads were removed by magnetic selection and the depleted phage were incubated with 10ug/mL of biotinylated HLA-HPV-E7 peptide complex in blocking buffer for 30. The antigen-phage complex was then pulled down by magnetic beads, washed 5 times with PBS-T (1X PBS + 0.05% Tween 20) followed by 3 washes with PBS alone. The bound phage was eluted, amplified and the whole process was repeated until sufficient enrichment of specific clones was achieved. Monoclonal phages were generated from the third round of panning and were analyzed by direct ELISA using an anti-M13 phage monoclonal antibody. Monoclonal phage supernatants that showed HPV-E7 p11-19/HLA-A2 complex-specific binding were selected for further screening on T2 cells pulsed with HPV-E7p11-19, WT1-RMF or HPV-AAAA peptide. Confirmed binders were sequenced using standard protocols.

### Engineering full length human IgG1 and BiTEs (bispecific T cell engagers)

The full-length IgG for 1B1, 2A5 and 3F8 was constructed by cloning variable heavy into human IgG1 constant domain and variable light into human kappa light chain backbone. The gene was cloned into pcDNA3.4 vector for expression in mammalian cells. For BiTE construction, 3F8 scFv in vL-vH was linked via G4S linker to an anti-CD3 L2K scFv [19] in vH-vL orientation. A 6xHis tag was added at the C-terminus of CD3 scFv to assist in purification. The gene was cloned into a pcDNA3.4 backbone as described above for mammalian cell expression.

### Expression and purification

Full length human IgG1 and 3F8 BiTE were expressed in Expi293F cells [Thermo Scientific, Inc.] as per manufacturer guidelines. Briefly, cells were transfected at 3x10^6^/mL with >95% viability at linear growth. Enhancers were added on the following day and culture was harvested on day 5. Media was cleared by centrifugation and filtering through a 0.22μm membrane. Cleared supernatants were processed for protein purification using either MabSelect SuRe [IgG] or His-trap excel resin (BiTE) (Cytiva, former GE healthcare).

### Characterization of the full-length hIgG1 for the HPV-E7p11/A2 complex

The specificities of the fully human IgG1 mAbs for the HPV-E7/A2 complex were determined by staining T2 cells pulsed with or without HPV-E7p11-19, WT1-RMF or HPV-E7AAAA peptides, after directly conjugating mAbs to APC. Binding of the BiTE was determined by staining cells with the 3F8 BiTE, followed by anti-His/PE secondary antibody. The fluorescence intensity was measured by flow cytometry. The same method was used to determine the binding of the mAbs to peptide un-pulsed tumor cell lines with endogenous expression.

### 3F8 BiTE-redirected T cell cytotoxicity

The 3F8-BiTE or its control BiTE at indicated concentrations were incubated with target cells and PBMCs at different effector: target [E/T] ratios for 5 hours. The cytotoxicity was measured by standard ^51^Cr-release assay. In brief, target cells were labeled with Na_2_^51^CrO_4_ [50 uCi/million cells] for one hour, washed and incubated with effector cells for 5 hours. The supernatant fluid was collected and radioactivity was measured in a gamma counter. Percentage specific lysis was determined from the following formula: 100 × [(experimental release − spontaneous release)/(maximum release − spontaneous release)]. Maximum release was determined by lysis of radiolabeled targets in 2.5% Triton X-100.

### Binding affinity of mAbs

Binding affinities of candidate antibodies in solution were determined using surface plasmon resonance (SPR) MASS-2 instrument [Bruker]. Biotinylated HLA-E7 peptide complex ligand (46 kDa) was immobilized on Biotin-Tag Capture Sensor (Bruker). Candidate antibodies (150 kDa) serially diluted in running buffer [1XPBST, pH7.2] starting at 1μM concentration were injected over the chip at 25°C. The complex was allowed to associate and dissociate for 180 and 300s, respectively. The data were processed using steady state 1:1 interaction model with double referencing.

### Isolation and sequence identification of HLA ligands by mass spectrometry (MS)

Cell lines were cultured until 10M to 30 million cells were available and then harvested. Harvested cells were pelleted and washed 3 times in ice-cold sterile PBS (Gibco, Cat 10010–0230.) Immunoprecipitation, HLA ligand separation and LC-MS/MS were performed as previously described [20]. Briefly, cells were lysed in 7.5 mL of 1% CHAPS (MilliporeSigma) for 1 hour at 4°C, lysates were spun down for 1 hour with 20,000*g* at 4°C, and supernatant fluids were isolated. For immunopurification of HLA class I ligands, 0.5 mg of W6/32 antibody (BioXCell) were bound to 40 mg CN-Br activated sepharose and incubated with the protein lysate overnight. HLA complexes and binding peptides were eluted five times using 1% TFA. Peptides and HLA complexes were separated using C18 columns (Sep-Pak C18 1 cc Vac Cartridge, 50 mg sorbent per cartridge, 37–55 μm particle size, Waters). C18 columns were preconditioned with 80% ACN (MilliporeSigma) in 0.1% TFA and equilibrated with 2 washes of 0.1% TFA. Samples were loaded, washed again twice with 0.1% TFA, and eluted in 300 μL of 30%, 40%, and 50% acetonitrile in 0.1% TFA. All three fractions were pooled, dried down using vacuum centrifugation and stored at -80°C until further processing. HLA ligands were isolated by solid-phase extractions using in-house C18 minicolumns. Samples were analyzed by high-resolution/high-accuracy liquid chromatography-tandem mass spectrometry (LC-MS/MS]) (Lumos Fusion, ThermoFisher Scientific). MS and MS/MS were operated at resolutions of 60,000 and 30,000, respectively. Only charge states 1, 2, and 3 were allowed. The isolation window was chosen as 1.6 thomson, and collision energy was set at 30%. For MS/MS, maximum injection time was 100 ms with an automatic gain control of 50,000. MS data were processed using Byonic software (version 2.7.84, Protein Metrics) through a custom-built computer server equipped with 4 Intel Xeon E5-4620 8-core CPUs operating at 2.2 GHz and 512 GB physical memory (Exxact Corporation). Protein FDR was disabled to allow complete assessment of potential peptide identifications. Oxidization of methionine; phosphorylation of serine, threonine, and tyrosine; as well as N-terminal acetylation were set as variable modifications for all samples. Samples were searched against a database comprising UniProt human reviewed proteins supplemented with the HPV proteome as well as common contaminants. For mirror plots ion intensities were exported and re-plotted with Graphpad prism.

## Results

### Selection of scFv specific for HPV-E7p11A2 complex and engineering of full-length human mAb

To select phage clones that mimic TCR recognition, e.g. recognizing amino acids in the middle of the peptide/HLA complex, we performed the following screening strategies: first, counter screening against a peptide/MHC complex with a peptide with a non-similar sequence to HPV using peptide RMFPNAPYL (from the WT1 protein) that binds to HLA-A2 with high affinity. WT1-RMF/HLA-A2 complex to remove all clones that bound to the HLA-A2 molecule. Second: positive screening for HPV-E7p11 (YMLDLQPET) /HLA-A2 complex to broadly select clones that bound to the desired HPV complex. Third: screening against a mutant peptide E7-AAAA [YMAAAAPET]/HLA-A2 complex to remove phage clones that bind to either end of the peptide. In addition, specificity to middle amino acids LDLQ should reduce binding to many potential human proteomic off-target peptides. Positive clones specific for the HPV E7p11/HLA-A2 complex were selected by 3–4 rounds of panning, confirmed by ELISA and live cell binding to T2 cells pulsed with HPV-E7p11 or other control peptides. Three out of total 19 phage clones were selected and engineered into full length human IgG1 format for further characterization.

**Fig 1:**
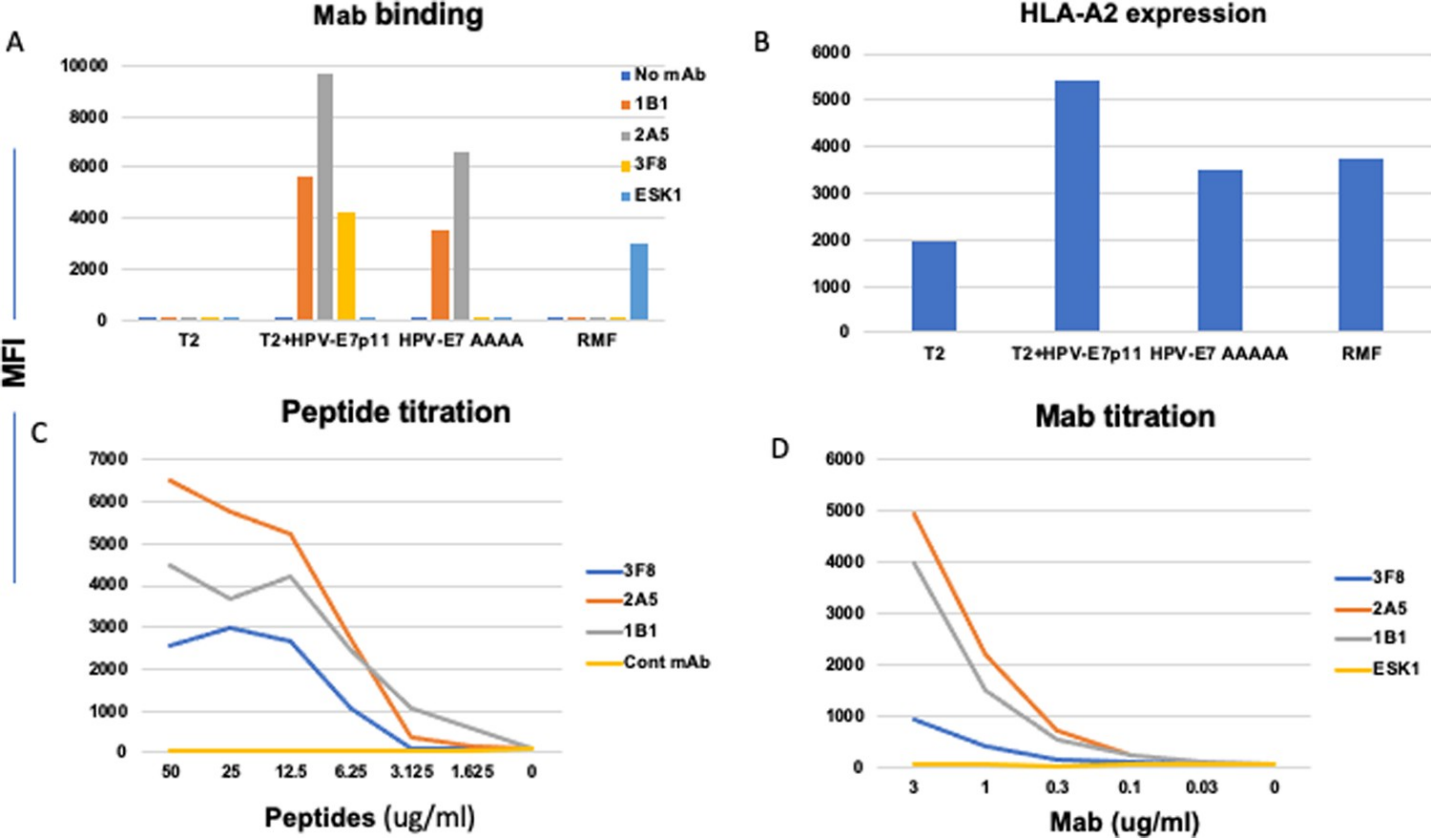
Binding of the mAbs to the HPV-E7p11-19/HLA-A2 complex. **[A]** Binding of mAbs to T2 cells pulsed with or without peptides. HPV-E7p11, HPV-E7AAAA or, WT1-RMF peptide at a concentration of 20ug/ml was pulsed onto T2 cells overnight in serum-free RPMI1640 complete medium. Cells were washed and stained with the mAbs 1B1, 2A5 or 3F8 conjugated to APC at a concentration of 3ug/ml. A control TCRm mAb specific for WT1-RMF/HLA-A2 complex, ESK1, was used as a negative control for HPV mAb, but positive assay control for RMF/HLA-A2 complex. In parallel, HLA-A2 expression stabilization was determined by staining the cells with anti-HLA-A2 mAb BB7 clone [B]. Binding potency of the mAbs was measured by titrating the HPV-E7p11-19 peptide at the indicated concentrations onto T2 cells; the cells were stained with indicated mAbs at 3ug/ml [C]. Mab titration was performed for relative avidity on T2 cells pulsed with HPV-E7p11-19 peptide at 20ug/ml and stained with the indicated mAbs at concentrations ranging from 3ug/ml to 0.03ug/ml] [D]. All binding was determined by flow cytometry and indicated by median fluorescence intensity [MFI].

The specificity of full length human IgG1 mAbs was evaluated using T2 cells, pulsed with or without HPV-E7p11, HPV-AAA or control peptide RMF, and tested for their binding capacity. All three hIgG mAbs, named 1B1, 2A5 and 3F8, bound to T2 pulsed with HPV-E7p11, but not to T2 cells alone, or pulsed with irrelevant RMF peptide. When T2 cells were pulsed with HPV-AAAA peptide, the 1B1 and 2A5 mAbs retained binding at a lower level, but 3F8 completely lost binding [Fig 1A]. These data show that 3F8, but not other two mAbs, was dependent on recognition of the middle amino acids, as planned, and similar to TCR binding. T2 stabilization assays showed that all tested peptides were sufficient to stabilize HLA-A2 on the cell surface and that the cell surface peptide-A2 expression was not confounding the specificity analysis [Fig 1B]. A comparison of the binding activity of the three mAbs specific for the HPV-E7p11/A2 complex was investigated by titration of both the peptide and the mAbs. 1B1 mAb showed the strongest affinity by recognizing the peptide below 1ug/ml, followed by 2A5 (between 3.12 and 1.625 ug/ml) and 3F8 (around 3.125ug/ml). [Fig 1C]. Antibody titration also confirmed that 3F8 was the weakest binder to the HPV/A2 complex among three mAbs [Fig 1D]. The affinity of the mAbs was determined by MASS-2 surface plasmon resonance instrument (Bruker); the affinity of 3F8 to HLA/peptide complex was low at 1.8uM, in contrast to 1B1 [30nM] and 2A5 [212nM] [Table 1], consistent with the binding data.

**Fig 2:**
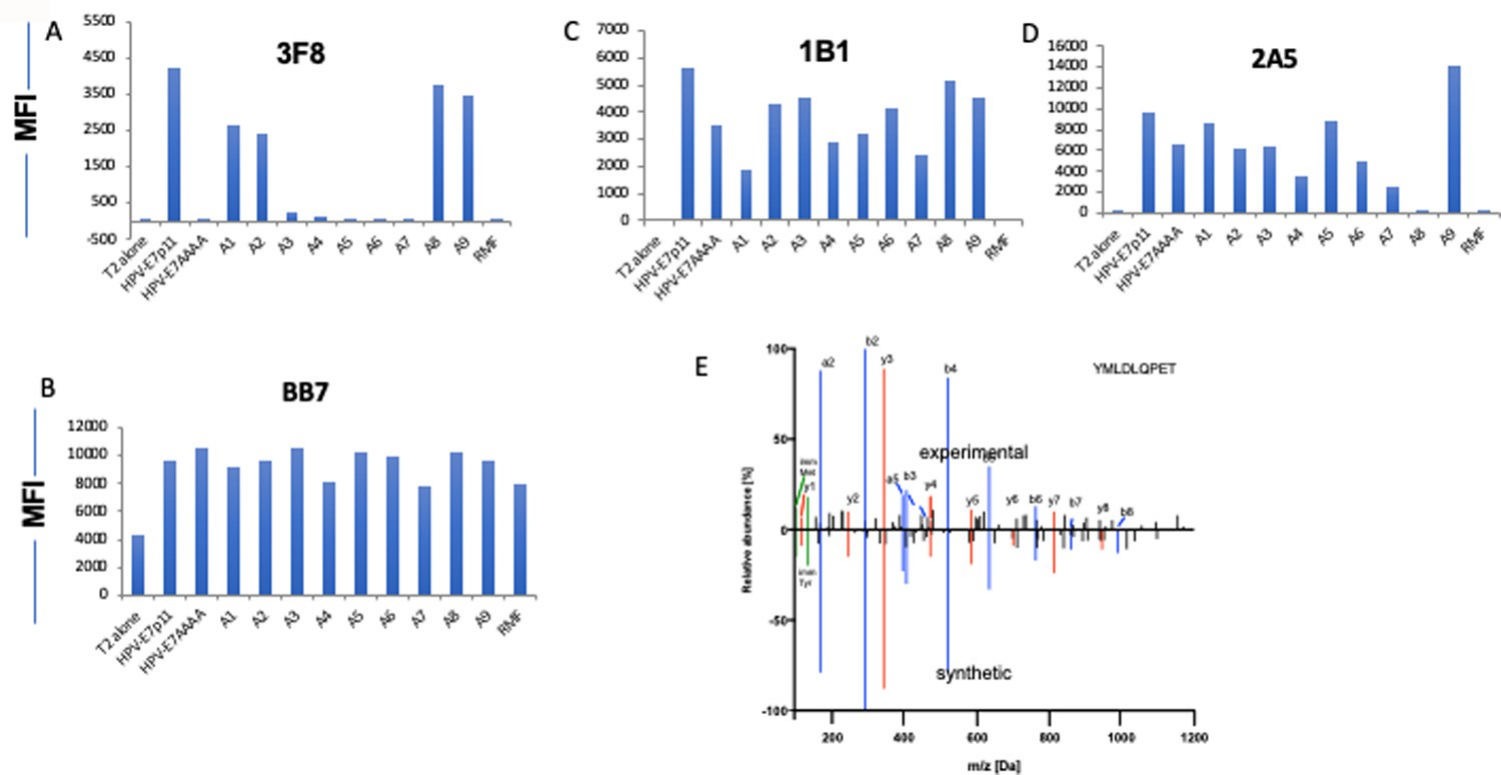
Epitope specificity. The target HPV-E7p11-19 peptide sequence or the same sequence substituted with alanine at positions 1, 2, 3, 4, 5, 6, 7, 8, 9 indicated as A1 to A9, respectively, and pulsed onto T2 cells at 20ug/ml. The HPV sequence with the 4 middle amino acids substituted with alanine [HPV-E7AAAA] as used to gage binding to the center of the sequence as well. The binding of the mAbs 3F8 [A], 1B1 [B], or 2A5 [C] at a concentration of 3ug/ml was determined by flow cytometric analysis. T2 cells alone, or pulsed with RMF irrelevant peptide were the negative controls. The same cells were simultaneously stained with anti-HLA-A2 mAb, clone BB7.2, to measure the relative binding of the peptides to HLA-A2 molecule [D]. The data represent one of two similar experiments. [E]. Mirror plot of synthetic [bottom] and cell-derived experimental [top] YMLDLQPET peptides. Relative abundance refers to peak areas which are normalized to maximum peak.

The binding positions of the mAbs were further investigated by pulsing T2 cells with HPV-E7p11 analog peptides that were substituted with alanine at each amino acid position [Table 2]. 1B1 and 2A5 binding ability was only partially reduced following substitutions at positions 1, 4, 5, 7, and 3, 4, 6, 7 and 8, respectively [Fig 2]. In contrast, 3F8 binding was completely abrogated when the HPV-E7p11 peptide was substituted with alanine at positions 3, 4, 5, 6, and 7. Differential binding to HPV-9AAAA peptide among the three mAbs further demonstrated that only 3F8 binds to the middle amino acids of the HPV-E7p11 peptide. In T2 stabilization assays, HLA-A2 expression was partially reduced with alanine substitution at position 4 and 7, [Fig 2B] that might have contributed to the reduced binding of the mAbs 1B1 and 2A5 [Fig 2C and 2D]. These data suggested that the mAb 3F8 recognition required most of the amino acids in the middle of the peptide and was thus more specific for the HPV-E7p11/HLA-A2 complex.

**Table 2:** Binding kinetics of antibodies to HLA-E7 peptide complex measured by SPR, as described in the Methods.

Antibody	k_on_ [1/Ms]	k_off_ [1/s]	K_D_ [nM]
1B1	1.19 × 10^4^	3.52 × 10^−4^	30
2A5	1.69 × 10^5^	2.26× 10^−7^	212
3F8	7.93 × 10^4^	1.81 × 10^−6^	1800

**Fig 3:**
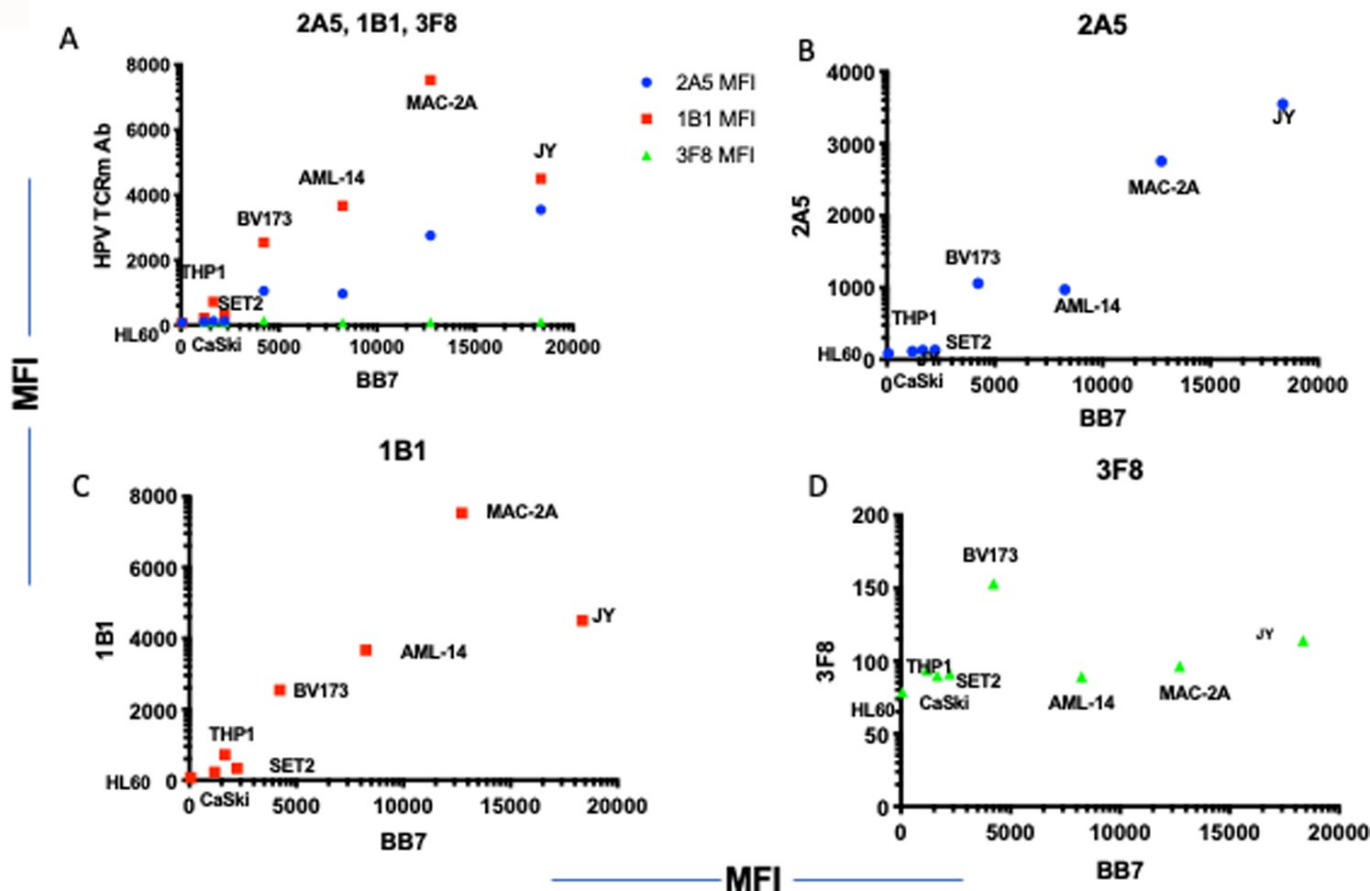
Correlation of HPV mAb binding and HLA-A2 expression. A panel of cell lines with variable amounts of HLA-A2 on the cell surface were stained with mAbs 1B1, 2A5 and 3F8 at 3ug/ml and BB7.2 to HLA-A2. HPV mAb binding is shown in Y-axis and HLA-A2 expression in X-Axis. [A] A comparison of all 3 mAb on the same plot. Each mAb alone is plotted: 2A5 [B], 1B1 [C], 3F8 [D]. The data is one representative from five independent experiments.

To further assess the specificity of the mAbs, a panel of HLA-A2-positive or negative cell lines [Table 3] that are either HPV positive or negative, were tested for the binding by the three mAbs. The presentation of the targeted HLA ligand YMLDLQPET on the cell surface of the cervical cancer cell line Caski, which had previously been reported to present the epitope [11], and other lines was confirmed using isolations of HLA ligands via immunoaffinity purification followed by mass spectrometry analysis. These experiments detected the targeted peptide exclusively in the Caski cell line and no other HPV-derived HLA ligands were identified [Table 3, Fig 2E]. The binding of both 2A5 and 1B1 mAb to the cell lines also was dependent primarily on the level of HLA-A2 expression; there was no binding to the HLA-A2 negative/HPV-negative AML cell line HL-60 [Fig 3A–3C]. 3F8 binding among the cell lines tested was low and independent of the level of HLA-A2 expression [Fig 3A and 3D]. Therefore, because of their lack of specificity, the 1B1 and 2A5 mAbs were excluded from further investigation.

**Table 3:** Cell lines used for mAb binding.

Cell line name	Cell origin	HLA-A*02:01 [by flow cytometry]	HPV-E7 mRNA	HPV-E7p11-19 epitope [From MS]
MAC2A	T cell lymphoma	Pos	Neg	NT
JY	EBV-positive B cell lymphoblastoid	Pos	NT	NT
BV173	CML/ALL	Pos	Neg	NT
AML-14	AML	Pos	NT	NT
SET2	AML	Pos	NT	NT
THP1	AML	Pos	NT	NT
Caski	Cervical cancer	Pos	Pos	Pos
Jurkat	T lymphoblastic leukemia	Neg	NT	NT
HL-60	CML	Neg	NT	NT

Pos: Positive; neg: Negative; NT: Not tested.

**Fig 4:**
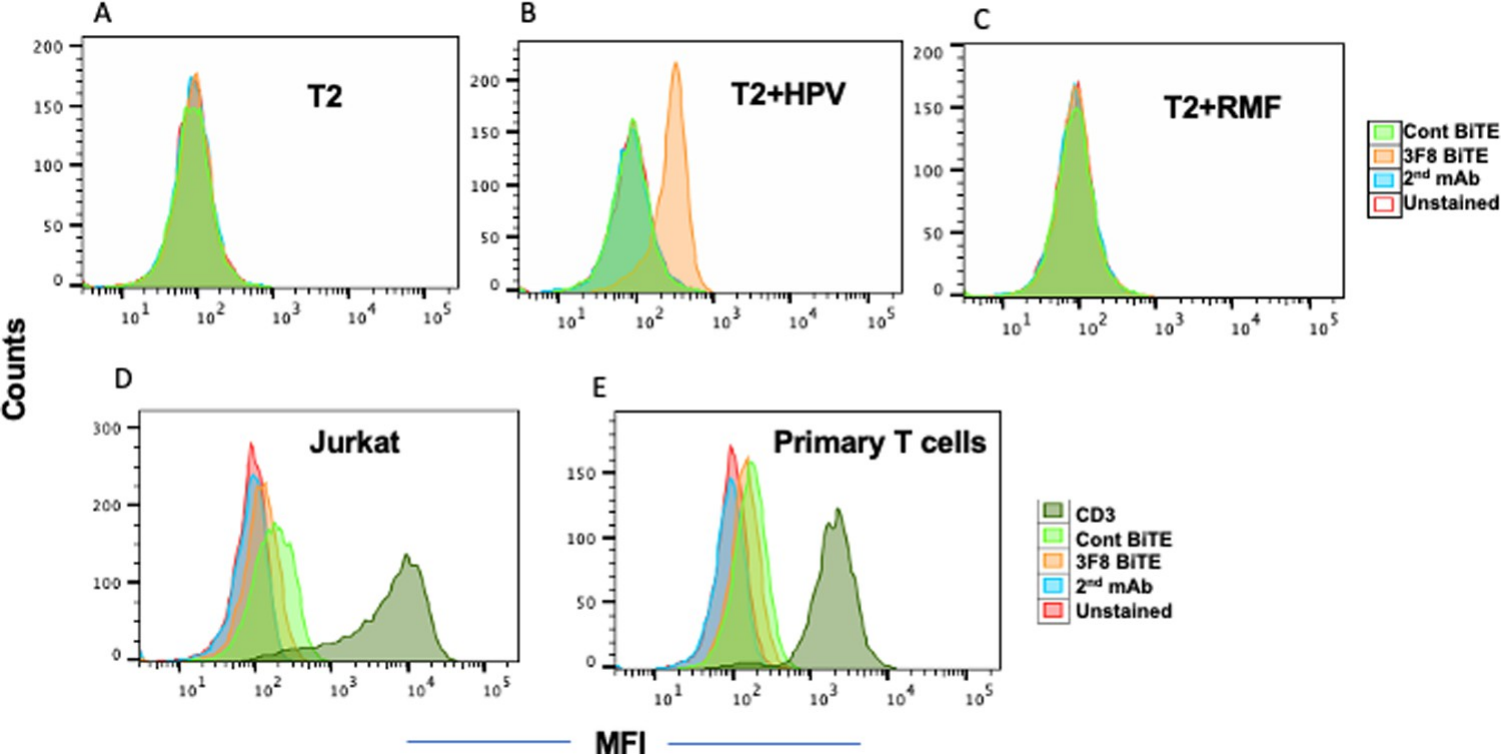
Specificity analysis of the 3F8 BiTE. Binding of 3F8 to HPV-E7p11/HLA-A2 complex. T2 cells alone [A] or pulsed with HPV-E7p11-19 [B], or RMF peptide [C] at a concentration of 20ug/ml overnight. Cells were washed and stained with 3F8 BiTE [3ug/ml], followed by secondary antibody to His-tag. Binding of 3F8 BiTE to cell surface CD3. Jurkat cells [D] or primary human T cells [E] were stained with 3F8 or control BiTE at a concentration of 3ug/ml, followed by anti-His-PE secondary antibody. A control anti-CD3 antibody was used to confirm the CD3 expression.

The low affinity of the mAb 3F8 suggested it might not have useful functional activity in an IgG format, especially for ultra-low density antigens such as HPV E7 PV HOpeptide/MHC complexes; therefore, we proceeded to engineer 3F8 to a bispecific T cell engager (BiTE) format to enhance its potency. Binding specificity of 3F8 BiTE for HPV-E7p11 was tested using T2 cells. There was no binding to either T2 alone or T2 when pulsed with RMF peptide, but the 3F8 BiTE bound well to T2 pulsed with the HPV target peptide [Fig 4A–4C]. For the other arm of the BiTE, CD3 recognition, both Jurkat (of T cell origin) and primary human T cells were tested. The 3F8 BiTE bound weakly to both cell types [Fig 4D and 4F].

Although BiTE recognition is monovalent for both the target and the T cell, this level of binding should be sufficient to elicit functional cytotoxicity. We next assessed the efficacy and potency of 3F8 BiTE-directed T cell cytotoxicity in 5 hr 51Cr- release assays against T2 cells pulsed with HPV or control RMF peptide. Compare to control BiTE, 3F8 BiTE showed cytotoxicity against T2 pulsed with HPV E7-p11-19, down to levels below 10ng/ml. These data demonstrated that 3F8, while a low affinity mAb, was able to kill the target with specificity.

**Fig 5:**
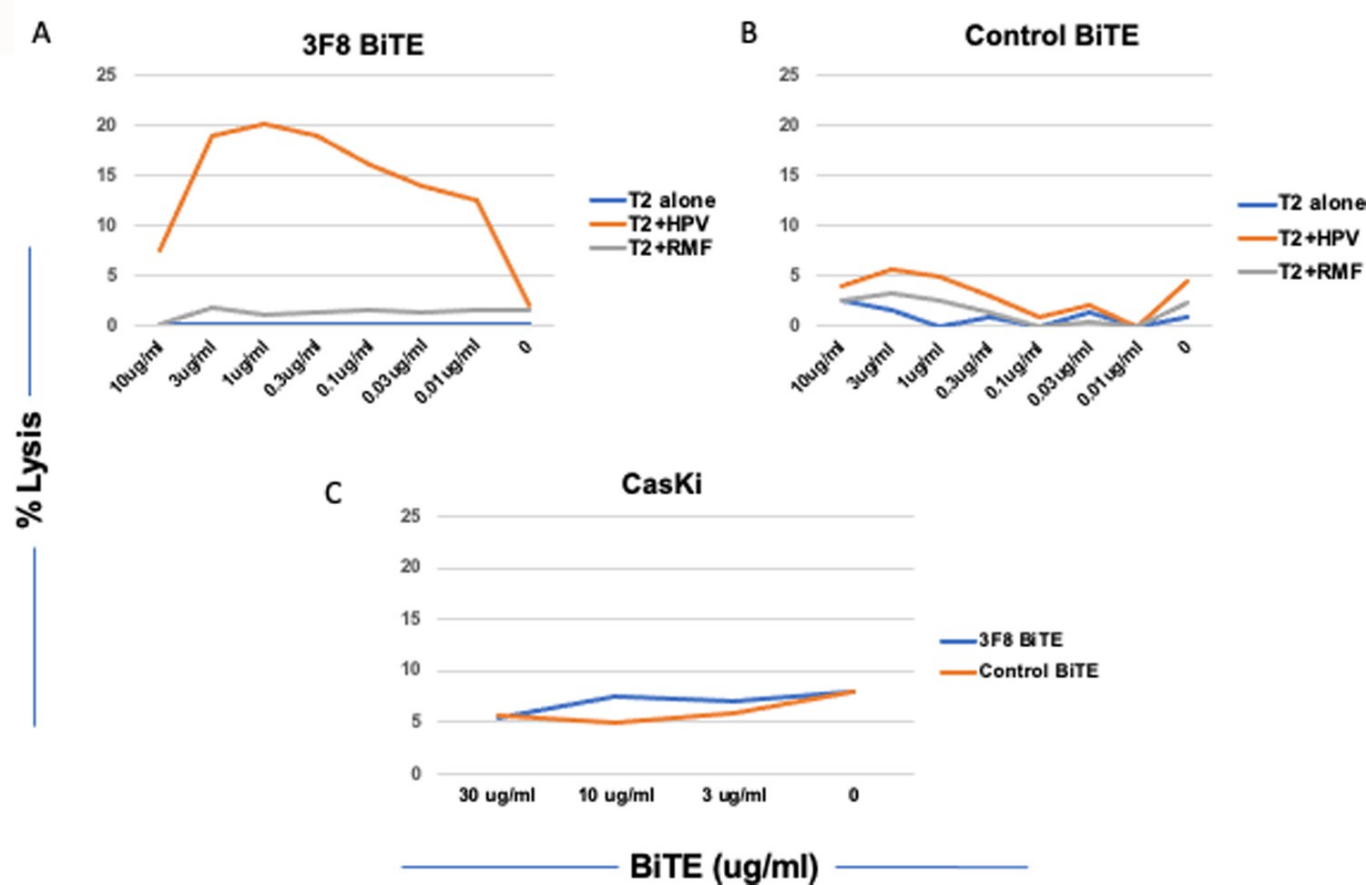
Cytotoxicity of the 3F8 BiTE. T2 cells alone, or pulsed with HPV-E7p11-19 or RMF control peptide at 50ug/ml were incubated with human PBMCs at E:T ratio of 20:1 and the 3F8 BiTE [Fig 5A], or control BiTE [Fig 5B] at indicated concentrations for 5 hrs and the cytotoxicity was measured by ^51^Cr-release assay. Similarly, 3F8 BiTE was tested for its cytotoxicity against CasKi [Fig 5C] without HPV E7-11 peptide pulsing at indicated concentrations. Each data point is the average of triplicate cultures and representative of three similar experiments.

To test if 3F8 mAb was able to recognize the naturally processed HPV-E7p11-20 epitope presented by HLA- A*02:01 molecules, multiple tumor cell lines that are HLA-A*02:01 and HPV-E7 positive derived from human cervical cancer were tested for the binding of the 3F8 hIgG1 and cytotoxicity of the 3F8-BiTE [Fig 5]. The BiTE format was used because of its higher potency than the Ig format. As noted above, one of the lines was confirmed to have the peptide epitope present on the cell surface complexed in HLA by mass spectrometry [Table 3]. However, 3F8 failed to bind or kill this mass spectrometry-positive tumor cell line Caski [Fig 5C]. These results suggest that the naturally presented antigenic density of the HPV-E7p11/HLA-A2 complex must be too low to allow. cell killing by a low affinity mAb, such as 3F8.

## Discussion

The development of TCRm mAbs is emerging as a powerful strategy to effectively target intracellular tumor antigens and expands the therapeutic opportunities for currently undruggable oncogenic and tumor-associated proteins. Virus-derived tumor antigens such as from HPV remain an attractive target because of their lack of normal tissue expression. The challenge for T cell-based therapies including TCRm mAbs remains the identification of suitable epitopes to ensure on-target specificity. While various HPV16-derived peptides have been tested as vaccines, the HPV-E7p11-19 was an epitope that was reproducibly detected by mass spectrometry analysis of HPV 16-E7-expressing cervical cancer cell lines and biopsies from patients with cervical cancer [12–14]. Therefore, HPV-E7p11-19 is a highly validated epitope for use in TCR based therapeutic strategies. In a recent study, a TCR specific for the E7p11-19/HLA-A2 was isolated from a patient with cervical cancer [11]. This TCR demonstrated high functional avidity. Human T cells, after transduction with the TCR gene, recognized and killed HPV16+ cervical and oropharyngeal cancer cell lines and mediated regression of established HPV16+ human cervical cancer tumors in a mouse model [11]. More recently, infusion of the T cells engineered with this TCR into patients with metastatic human HPV-associated cancers, induced robust tumor regression with objective clinical responses in 6 of 12 patients, including 4 of 8 patients with anti-PD1 refractory disease [21]. Clinical responses included regression of bulky tumors and complete tumor regressions in some patients. These studies further validated the HPV-E7p11 epitope presented by HLA-A2 as clinically useful and indicated it also could be a potential therapeutic target for a TCRm mAb approach.

Here, we show the discovery of the first TCRm mAb specific for the HPV16-E7p11 epitope when presented by HLA-A2 on the cell surface. Our initial characterization has demonstrated the specificity and the functional activity of a mAb clone, 3F8. Alanine scanning showed a broad range of amino acids from positions 3–8 that were important for the recognition by the mAb 3F8. This predicts a low risk for broad cross-reactivity to similar off-target amino acid sequences that may be in the proteome. A BiTE form of 3F8 was also able to kill the T2 cells pulsed with the target peptide, but not irrelevant peptide, despite the low affinity of the mAb. This could be due to an ability of the BiTE engaging a serial killing of the target cells once T cells are activated [22]. However, the effector function of 3F8 was only limited to T2 cells pulsed with target peptide but not to tumor cells lines that are positive for both HPV-E7 and HLA-A2 and had the peptide documented as present by mass spectrometry. This could be explained by both the low affinity of 3F8 [1.8uM] and low density of the HPV-E7p/HLA-A2 complex on the cell surface. HPV-E7p11/HLA-A2 complexes have been shown to be found at 25 copies per cell of Caski [11], the line that was target in this report. It is well known that both antibody affinity and antigenic density on cell surface are driving factors for the specificity and the effector function of the mAbs. Mabs in various soluble forms targeting cell surface protein targets [10,000 to 1,000,000 molecules per cell] with picomolar to nanomolar affinities have functional cytolytic activities such as ADCC and complement-dependent cytotoxicity [CDC] [23,24]. In contrast, peptide/MHC complexes are ultra-low density antigens [with tens to hundreds of copies per cell] unlike to most mAb-targeted proteins on the cell surface. Functional TCRm mAbs reported to date have affinities in nanomolar range [25,26]. To overcome these obstacles, antibody affinity maturation could be considered to increase the affinity if it can be achieved without compromising the mAb specificity [27]. A variety of different Ig isotypes might be attempted to activate other effectors such as NK cells, macrophages, or mast cells. Alternatively, more potent formats of engineered bispecific mAbs have been developed to bridge powerful T cell cytotoxicity to the targets [28,29]. In particular, bispecific molecules directed against targets in low abundance like MHC presenting specific epitopes, for example mutant P53 and KRAS, required extremely high potency to be effective [30,31]. In a recent study, Douglas et al. generated various formats of TCR mimic bispecific mAbs specific for the RAS mutant epitope in the context of HLA. A single chain diabody (scDb) format mediated specific killing of cancer cells displaying only a few cell surface epitopes [32]. The ability to induce target lysis at such low antigen density is encouraging and it suggests that with appropriate bispecific mAb formats, it would be possible to target difficult ultra low-density antigens such as peptide/MHC complexes described here. The authors speculated that in contrast to BiTE, which contains a single flexible linker between two scFvs, the scDb has a compact structure, that may have limited flexibility between the antigen and CD3. This could form a tighter immunological synapse, resulting in more effective T cell activation. Since there have been limited studies using bispecific mAbs formats for TCRm mAbs, the mechanisms underlying the efficacy of BisAbs for p/MHC antigens are of particular importance, that remains to be further studied. Finally, such a low affinity mAb could be engineered into chimeric antigen receptor (CAR) T cell construct to increase the avidity and potency [33]. In conclusion, while more work is required to further improve the binding affinity of 3F8, and to reformat into a more potent agent to reach a desired functional properties, this study opens the possibility of targeting HPV-associated malignancies by use of TCRm mAbs.
